# Respiratory Muscle Training in Patients with Obstructive Sleep Apnoea: A Systematic Review and Meta-Analysis

**DOI:** 10.3390/clockssleep4020020

**Published:** 2022-04-11

**Authors:** Rodrigo Torres-Castro, Lilian Solis-Navarro, Homero Puppo, Victoria Alcaraz-Serrano, Luis Vasconcello-Castillo, Jordi Vilaró, Roberto Vera-Uribe

**Affiliations:** 1 Department of Physical Therapy, Faculty of Medicine, University of Chile, 8380453 Santiago, Chile; klgorodrigotorres@gmail.com (R.T.-C.); klga.solis@gmail.com (L.S.-N.); homeropuppo@gmail.com (H.P.); l.vasconcello.c@gmail.com (L.V.-C.); 2 Institut d’Investigacions Biomèdiques August Pi i Sunyer (IDIBAPS), 08036 Barcelona, Spain; 3 Barcelona Institute for Global Health (ISGlobal), 08003 Barcelona, Spain; victoriaalcarazserrano@gmail.com; 4 Blanquerna School of Health Sciences, Universitat Ramon Llull, 08025 Barcelona, Spain; 5 Blanquerna School of Health Sciences, Global Research on Wellbeing (GRoW), Universitat Ramon Llull, 08025 Barcelona, Spain; jordiVC@blanquerna.url.edu

**Keywords:** respiratory muscle training, obstructive sleep apnoea, apnoea/hypopnea index, sleepiness, sleep quality

## Abstract

Background: Effective treatments for obstructive sleep apnoea (OSA) include positive pressure, weight loss, oral appliances, surgery, and exercise. Although the involvement of the respiratory muscles in OSA is evident, the effect of training them to improve clinical outcomes is not clear. We aimed to determine the effects of respiratory muscle training in patients with OSA. Methods: A systematic review was conducted in seven databases. Studies that applied respiratory muscle training in OSA patients were reviewed. Two independent reviewers analysed the studies, extracted the data and assessed the quality of evidence. Results: Of the 405 reports returned by the initial search, eight articles reporting on 210 patients were included in the data synthesis. Seven included inspiratory muscle training (IMT), and one included expiratory muscle training (EMT). Regarding IMT, we found significant improvement in Epworth sleepiness scale in −4.45 points (95%CI −7.64 to −1.27 points, *p* = 0.006), in Pittsburgh sleep quality index of −2.79 points (95%CI −4.19 to −1.39 points, *p* < 0.0001), and maximum inspiratory pressure of −29.56 cmH_2_O (95%CI −53.14 to −5.98 cmH_2_O, *p* = 0.01). However, the apnoea/hypopnea index and physical capacity did not show changes. We did not perform a meta-analysis of EMT due to insufficient studies. Conclusion: IMT improves sleepiness, sleep quality and inspiratory strength in patients with OSA.

## 1. Introduction

Obstructive sleep apnoea (OSA) is a breathing disorder characterised by narrowing of the upper airway that impairs normal ventilation during sleep [1]. OSA affects between 9% and 38% of the adult population, constituting a public health concern, particularly in overweight and obese subjects [2]. This disease is associated with many health consequences, including daytime sleepiness, metabolic and cardiovascular diseases and cognitive impairment [3]. In addition, OSA is strongly associated with cerebrovascular disorders, chronic neurodegenerative and inflammatory diseases, leading to a high risk of cognitive impairment in affected patients [4].

The “gold standard” treatment for OSA is continuous positive airway pressure (CPAP), being the first choice in moderate or severe cases [5]. However, CPAP adherence is often low, as patients experience it as intrusive and challenging to wear throughout the night [6].

Other effective treatments include weight loss, oral appliances that hold the jaw forward during sleep, surgical modification of the pharyngeal soft tissues (i.e., lateral pharyngoplasty or uvulopalatopharyngoplasty) or facial skeleton to enlarge the upper airway, and exercise [5,7]. In addition, in recent years, comprehensive treatments that include general or specific muscle training have been suggested to lower the apnoea-hypopnea index (AHI) of the OSA patient population [8,9,10].

Respiratory muscle training (RMT) strengthens the inspiratory and/or expiratory muscles. This training is performed by breathing against a specific resistance through an adjustable valve [11]. In many conditions, such as respiratory or cardiovascular diseases, this type of training has shown significant results and improvements in crucial outcomes, such as functional capacity or symptoms [12,13,14].

There are multiple respiratory muscles involved in maintaining upper airway patency in persons anatomically predisposed to obstructive sleep-disordered breathing [15]. Additionally, the oropharynx is highly collapsible, and most individuals have a predisposition to the sleep-related collapse of the upper airway [8,15].

Although the involvement of the respiratory muscles in OSA is evident, the effect of RMT to improve clinical outcomes is not clear [16,17]. Therefore, we aimed to determine the effects of RMT in AHI and subjective symptoms in patients with OSA.

## 2. Methods

### 2.1. Protocol and Registration

We performed a systematic review according to the Preferred Reporting Items for Systematic Reviews and Meta-Analyses (PRISMA) guidelines [18]. The review was registered in the International Platform of Registered Systematic Review and Meta-analysis Protocols-INPLASY202220096.

### 2.2. Criteria for Considering Studies in This Review

We included randomised controlled trials (RCTs) of patients with a confirmed diagnosis of OSA. The included studies aimed to determine the effects of RMT in OSA patients. The search strategy was based on the PICO model (population: adults with OSA; intervention: RMT; control: no intervention or placebo; and outcome: apnoea/hypopnea index, sleepiness, sleep quality, physical capacity, respiratory muscle strength).

### 2.3. Search Strategies and Data Resources

We reviewed the Embase, PubMed/MEDLINE, Web of Science, CINAHL, Cochrane Register of Clinical Trials (CENTRAL), Scopus, and Scielo databases on 22 February 2022. We conducted manual searches with the following terms: ((inspiratory muscle training) OR (respiratory muscle training) OR (expiratory muscle training)) AND ((sleep apnoea) OR (sleep-disordered breathing)). We imposed no language or publication restrictions.

The terms selected were combined using Boolean logical operators (OR, AND, NOT). We also conducted a manual search of the references included in the selected articles. All references were analysed using Rayyan web software [19].

### 2.4. Reviewing Procedure and Data Extraction

The selected articles were reviewed independently by investigators with experience in meta-analysis and training in literature review. First, the titles and abstracts of all identified studies were reviewed by two investigators (RTC, LSN). Studies deemed not relevant based on the title and abstract review were excluded. Any disagreements were solved by a third reviewer (HP). Second, the full-text versions of the articles selected in the first step were read and rechecked against the eligibility criteria (RTC, LSN). Again, any disagreements were solved by a third reviewer (HP). Finally, additional unpublished data were obtained from study authors when possible.

### 2.5. Methodological Quality Assessment

An assessment of the methodological quality of the primary articles was carried out using the Cochrane Collaboration tool for assessing the risk of bias (the Cochrane Handbook for Systematic Reviews of Interventions) [20]. The tool included seven items: generation of a random sequence, allocation concealment, blinding of participants and personnel, blinding of outcome assessment, completeness of outcome data, selectivity of reports and other biases. For each item, the risk of bias for the study was rated according to three categories: low, high or unclear risk of bias. Two reviewers (RTC-LSN) independently assessed the risk of bias of the studies. A third author (HP) was consulted for discrepancies that could not be resolved.

### 2.6. Data Synthesis and Analysis

We reported summaries of the association between the outcomes for each study in terms of mean differences (MD) or standard mean differences (SMD) using Review Manager 5 (RevMan, Copenhagen: The Nordic Cochrane Centre, The Cochrane Collaboration, 2014). We compared absolute values and obtained combined measures of the effect of each primary outcome through meta-analysis with a random-effect model due to the expected heterogeneity between studies [20]. Statistical heterogeneity was measured with the I^2^ statistic and classified as low (I^2^ < 25%), moderate (I^2^ = 25–50%), or high (I^2^ > 50%) [20].

## 3. Results

### 3.1. Study Selection

**Figure 1:**
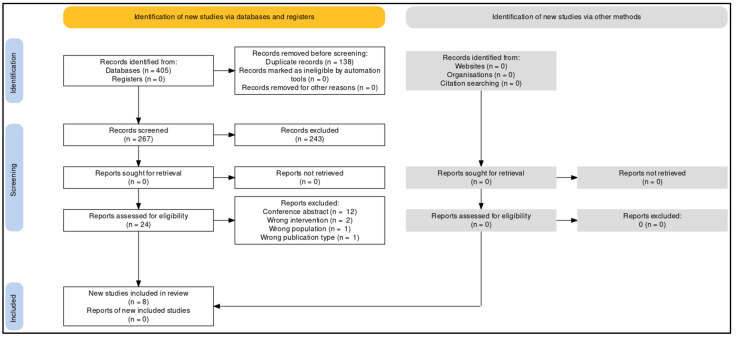
Study selection process.

The initial search yielded 405 potential studies. In total, 138 duplicate records were deleted. We screened 267 titles and abstracts and excluded 243 records that did not meet our inclusion criteria. Twenty-four of these were assessed as full-text. Of these, 12 were excluded for conference abstract, two for wrong intervention, one for wrong population, and one for wrong publication type. Ultimately, eight studies met the criteria for eligibility and were included in the review [16,17,21,22,23,24,25,26]. The flow chart of the study selection process is shown in Figure 1.

### 3.2. Characteristics of the Included Studies

Two studies were conducted in the USA [17,26], two in Taiwan [23,25], two in Brazil [16,21], one in Egypt [22], and one in Turkey [24]. All studies were published after 2016. The characteristics of the included studies are shown in Table 1. Seven studies included IMT [16,17,21,22,23,24,26], and one applied expiratory muscle training (EMT) [25].

### 3.3. Participants

In total, 210 patients with OSA were analysed (115 in the intervention group and 95 in the control group). Sample sizes varied between 16 [16,21] and 55 [22] participants. The studies included 63 (30%) females and 147 (70%) males with mean ages varying between 44.3 ± 2.9 [25] and 69.7 ± 3.4 [17] years. The body mass index (BMI) varied between 24.7 ± 0.8 [25] and 33.4 (30.3–34.5) [16] kg/m^2^. The AHI varied between 14.6 ± 1.5 [25] and 38.7 ± 24.0 [24] events/h. One study did not report the BMI [21], and one did not report the AHI [26] (Table 1).

### 3.4. Characteristics of Training

Seven of the selected articles applied IMT [16,17,21,22,23,24,26] and one applied EMT [25] for the intervention. In the case of IMT, the devices used were Powerbreathe K3 (HaB International, Southam, Warwickshire, UK) [17,26], Powerbreathe Classic (HaB International) [16,21], IMT Threshold (Philips Respironics, Murrysville, PA, USA) [23,24] and TRAINAIR (Project Electronics Ltd., London, UK) [22]. The load used varied between 30% [23,24] and 75% [16,17,22,26] of the maximum inspiratory pressure (MIP). The duration of the programs varied between six [17,26] and 12 weeks [21,22,23,24]. Five articles trained for number of repetitions [16,17,21,22,26] and two for time [23,24]. In the case of EMT, the only article selected used an EMST150 device (Aspire Products, Gainesville, FL, USA) with a 75% load, 25 breaths/day for five weeks [25]. The detail of the training programs is shown in Table 2.

### 3.5. Methodological Quality Assessment

**Figure 2:**
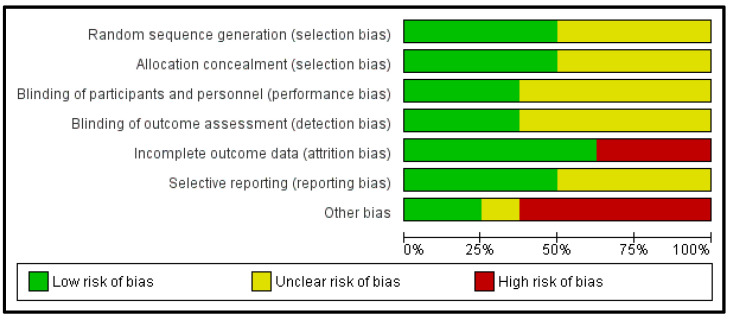
Risk of bias graph.

**Figure 3:**
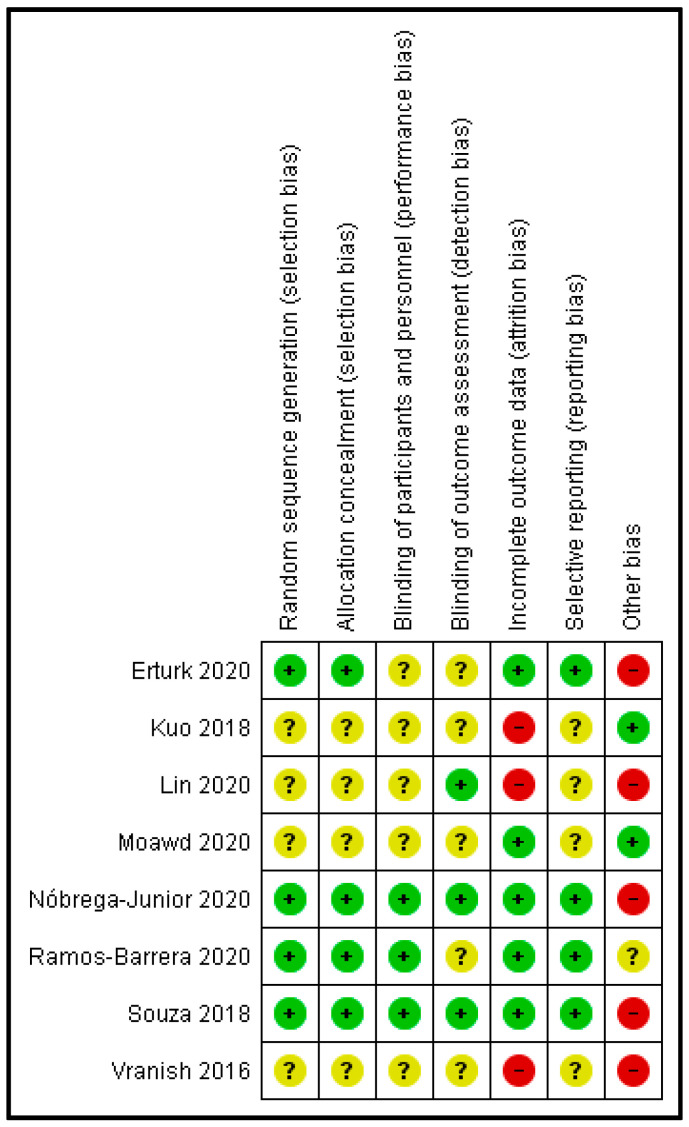
Risk of bias summary.

All studies had a high or unclear risk of bias in at least one domain. The majority of studies claimed to be randomised. However, only half of them explain how the randomisation was done [16,21,24,26]. Three studies reported that participants and personnel were blinded [16,21,26]. Three studies reported that researchers and outcome assessments were blinded [16,21,23]. Two studies had insufficient data on attrition rates [23,25]. Four studies had a low risk of selective reporting [16,21,24,26]; only two studies had a high risk of selective reporting. Finally, four studies had a high risk of other potential sources of bias due to poor participant compliance, sample size or baseline differences [16,17,21,23,24] (Figure 2 and Figure 3).

### 3.6. Main Findings

**Figure 4:**
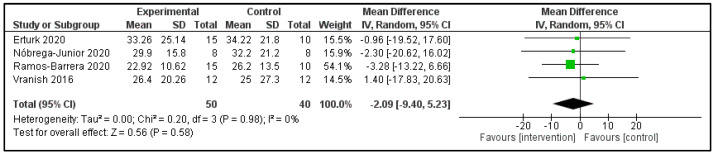
Forest plot for apnoea/hypopnea index.

**Apnoea/hypopnea index:** Four studies reported the AHI post-intervention (Table 2) [16,17,24,26]. These studies compared 50 patients in the intervention group (IG) versus 40 in the control group (CG). Both groups had similar AHI (MD −2.09 events/h; 95%CI −9.40 to 5.23 events/h; *p* = 0.58). The heterogeneity of the comparison was low (I^2^ = 0%) (Figure 4). The sole study of EMT showed that the reduction in the AHI of the IG (−40% ± 6%) was significantly greater than the CG (4% ± 6%; *p* < 0.05) [25].

**Figure 5:**
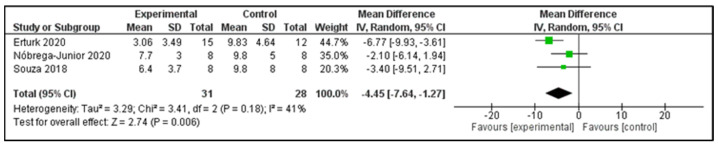
Forest plot for Epworth sleepiness scale.

**Sleepiness:** Three studies examined the Epworth sleepiness scale (ESS) post-intervention (Table 2) [16,21,24]. These studies compared 31 participants in the IG versus 28 participants in the CG. The heterogeneity of the comparison was moderate (I^2^ = 41%). Patients in the IG had, on average, −4.45 points (95%CI −7.64 to −1.27 points) of ESS in comparison to CG (*p* = 0.006) (Figure 5). The only study of EMT showed that the change of ESS scores did not differ between the IG and CG [25].

**Figure 6:**
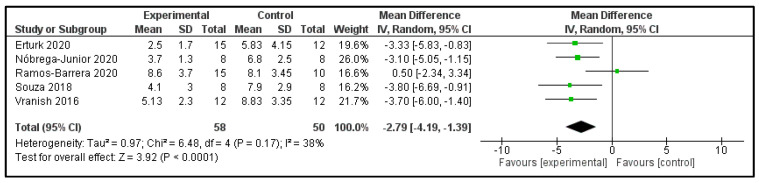
Forest plot for Pittsburgh sleep quality index.

**Sleep quality:** Five studies examined the Pittsburg sleep quality index (PSQI) post-intervention (Table 2) [16,17,21,24,26]. These studies compared 58 participants in the IG versus 50 participants in the CG. The heterogeneity was moderate (I^2^ = 38%). Patients in the IG had −2.79 points (95%CI −4.19 to −1.39) in comparison to CG (*p* < 0.0001) (Figure 6). The only study of EMT showed that the score of the IG (−28% ± 5%) improved significantly more than did those of the CG (10% ± 14%; *p* < 0.05) [25].

**Figure 7:**
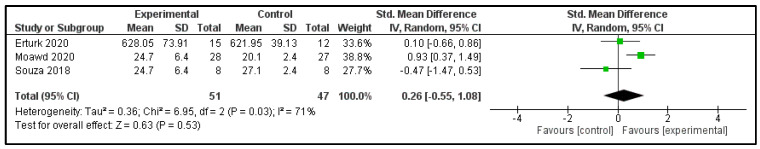
Forest plot for physical capacity.

**Physical capacity:** Three studies reported physical capacity post-intervention (Table 2) [21,22,24]. The outcomes reported were VO2_peak_ [21,22]_,_ and the distance walked in the six-minute walk test [24]. These studies compared 51 patients in the IG versus 47 patients in CG. Both groups had similar values (SMD 0.26; 95%CI −0.55 to 1.08; *p* = 0.53). The heterogeneity of the comparison was high (I^2^ = 71%) (Figure 7).

**Figure 8:**
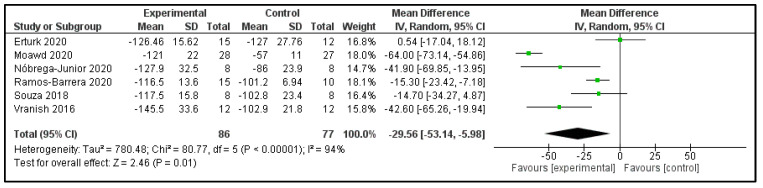
Forest plot for maximum inspiratory pressure.

**Inspiratory muscle strength:** Six studies examined the MIP post-intervention (Table 2) [16,17,21,22,24,26]. These studies compared 86 participants in the IG versus 77 in the CG. The heterogeneity was high (I^2^ = 94%). Patients in the IG had, on average, −29.56 cmH_2_O (95%CI −53.14 to −5.98 cmH_2_O) in comparison to CG (*p* = 0.01) (Figure 8).

## 4. Discussion

Our results found that IMT improves sleepiness, sleep quality and MIP in OSA patients; however, the AHI and physical capacity did not show changes.

The main index that guides OSA treatment is the AHI [5]. However, our results show that it is not modifiable with IMT. Other interventions, such as general physical exercise, decreased the AHI [27]. While the mechanisms underlying these beneficial effects in OSA patients are not fully understood, it is known that exercise can reduce body mass and fat mass, which have been related to significant reductions in the AHI [8]. However, these effects are difficult to achieve with specific training for a relatively small muscle group.

Although there was no change in the AHI, there were significant improvements in sleepiness. Moreover, the change was clinically significant because the minimal clinically meaningful improvement in the ESS is between −2 and −3 [28]. The ESS is commonly used to examine self-reported daytime sleepiness in clinical populations; however, the physiological correlate of this scale is not well understood [29]. In addition, the literature has shown that standard measures of both usual sleep length and timing and PSG measures of a single night of sleep are poor predictors of ESS scores [29]. For this reason, it is not surprising that there is a divergence between the ESS and the AHI.

Sleep quality is a concept that includes quantitative aspects of sleep and more subjective aspects, such as “depth” or “restfulness” of sleep [30]. The most used instrument is PSQI, an index created in the psychiatric field [30]. The minimal clinically significant improvement in PSQI is −3 [31], and we observed a change of −2.79, being not clinically relevant. As well as ESS, there is a difference in AHI results. It is important to say those discrepancies between objective and subjective sleep measures have diagnostic value for some sleep disorders.

We only found one study that analyses EMT [25]. This article demonstrated that EMT effectively improved sleep apnoea, sleep quality and expiratory muscle strength in participants with OSA [25]. Additionally, participants with moderate OSA exhibited greater improvement than those with mild OSA, and the improvement in MEP scores was correlated with a decrease in sleep apnoea [25]. A possible explanation is that expiratory muscle strength is more important than inspiration in overcoming upper airway obstruction [24]. The expiration is passive in the normal airway during calm breathing. When the airway resistance is increased, calm expiration cannot be performed, and active expiration is conducted using expiratory muscles (abdominal and internal intercostal muscles) to overcome the resistance to airflow [32].

Previous meta-analyses have reported the effect of interventions related to physical exercise in patients with OSA [33,34]. Aiello et al. showed that physical exercise has an effect on reducing both AHI and ESS in patients with OSA [33]. This conclusion remained consistent independent of different types of exercise, duration and frequency of exercise, CPAP usage, and supervised or unsupervised treatment programs [33]. In contrast to Aiello et al., we only explored the effect of RMT.

On the other hand, Cavalcante-Leão et al. and Hsu et al. conducted two meta-analyses of RMT [34,35]. They suggested that breathing exercises improve AHI for mild to moderate OSA patients, improving sleep quality and daytime sleepiness [34,35]. The eligibility criteria could explain the difference. Unlike both previously mentioned articles, our group excluded studies that did not include MIP-based programmable devices since these allow compliance with the principles of specificity and progressive overload training [36].

As with other diseases, the load, frequency of training and duration continue to be a matter of discussion. Although most of the studies of RMT follow the physiological principles of overload, specificity, and variability, there is a wide heterogeneity in the training programmes. Additionally, adherence was scarcely reported [23]. Although some authors describe high adherence rates, we cannot analyse the adherence [26].

Unlike other chronic respiratory pathologies, in which physical capacity improves after IMT [37,38,39], there were no significant changes in patients with sleep apnoea. A possible explanation for this non-effect is that the other pathologies have an additional crucial systemic commitment to the respiratory muscles. However, this seems not to be so important in patients with OSA who, despite the disease, do not show significant disability and continue to study or work without problems once they use CPAP.

Although this meta-analysis explored the effects on AHI and symptoms, IMT could have an effect at the cardiovascular level. Vranish and Bailey [17] found that subjects with OSA who performed IMT manifested reductions in systolic and diastolic blood pressures and plasma norepinephrine levels. These favourable outcomes were achieved without affecting AHI [17].

Our study has some limitations. First, the selected studies are few and do not allow for a sub-analysis according to OSA severity or training loads. Second, a common feature of the studies is that they have small sample sizes. Third, the heterogeneity of some outcomes was high (physical capacity and MIP), so these results should be analysed with caution. Finally, we found only one study with EMT, so we could not perform a meta-analysis with this type of training. Nevertheless, future research in this field should explore the effect of EMT, given the promising results shown by the only article that used this training modality.

## 5. Conclusions

IMT improves sleepiness, sleep quality and inspiratory strength in patients with OSA. Future studies are recommended in order to explore the benefits of EMT in OSA patients.

## Figures and Tables

**Table 1:** Characteristics of included studies.

Author, Year	Country	Group, *n*	Gender (M/F)	Age (Years)	BMI (kg/m^2^)	AHI (Events/h)	ESS	MIP (cmH_2_O)	MEP (cmH_2_O)
Vranish and Bailey, 2016	USA	IMT: 12	8/4	61.5 ± 3.9	27.0 ± 1.0	21.9 ± 4.4	NR	80.7 ± 7.1	NR
		Placebo: 12	8/4	69.1 ± 3.4	28.5 ± 1.6	29.9 ± 8.9	NR	75.2 ± 3.9	NR
Kuo et al., 2017	Taiwan	EMT: 13	11/2	44.3 ± 2.9	24.9 ± 0.5	16.5 ± 2.2	9.8 ± 1.1	NR	134.8 ± 10.4
		Control: 12	10/2	48.0 ± 3.1	24.7 ± 0.8	14.6 ± 1.5	9.8 ± 0.9	NR	108.6 ± 11.6
Souza et al., 2018	Brazil	IMT: 8	4/4	54.8 ± 6.9	NR	27.6 ± 11.9	11.1 ± 4.5	85 ± 23.5	130.3 ± 35.8
		Placebo: 8	6/2	49.9 ± 11.6	NR	34.0 ± 18.4	11.1 ± 6.8	87.1 ± 23.7	115.4 ± 29.1
Erturk et al., 2020	Turkey	IMT: 15	9/6	49.7 ± 9.1	31.0 ± 5.4	30.0 ± 19.3	8.9 ± 4.4	80.9 ± 16.9	120.5 ± 21.3
		Control: 12	10/2	47.3 ± 7.3	32.1 ± 3.7	38.7 ± 24.0	9.7 ± 5.9	131.7 ± 23.5	148.9 ± 32.3
Lin et al., 2020	Taiwan	IMT: 16	13/3	47.9 ± 12.2	26.2 ± 3.3	29.0 ± 2.8	10.5 ± 5.7	NR	NR
		Control: 6	5/1	56.2 ± 11.5	27.3 ± 3.6	37.5 ± 14.1	13 ± 2.6	NR	NR
Moawd et al., 2020	Egypt	IMT: 28	20/8	55.5 ± 9.8	29.2 ± 3.9	32 ± 11.7	NR	56 ± 13	NR
		Placebo: 27	22/5	59.5 ± 4.8	27.9 ± 4.8	31 ± 10.8	NR	52 ± 10	NR
Nóbrega-Júnior et al., 2020	Brazil	IMT: 8	3/5	58.6 ± 5.6	33.4 (30.3–34.5)	31.7 ± 15.9	12.5 ± 4.0	83.6 ± 26.5	124.8 ± 46.7
		Placebo 8	1/7	60.1 ± 2.7	32.7 (23.8–34.9)	31.4 ± 20.8	14.9 ± 5.2	74.6 ± 25.4	101.6 ± 29.4
Ramos-Barrera et al., 2020	USA	IMT: 15	11/4	65.9 ± 6.0	30.7 ± 6.2	NR	NR	82.6 ± 12.5	NR
		Control: 10	6/4	69.7 ± 6.7	31.3 ± 6.5	NR	NR	85.6 ± 4.5	NR

Abbreviations: BMI: Body mass index; AHI: Apnoea/hypopnea index; EMT: Expiratory muscle training; ESS: Epworth sleepiness score; MIP: Maximum inspiratory pressure; MEP: Maximum expiratory pressure; IMT: Inspiratory muscle training; NR: Not reported.

**Table 2:** Characteristics of respiratory muscle training programs.

Author, Year	Device	Load	Comparison	Frequency	Duration
Vranish J and Bailey F, 2016	K3 series, POWERbreathe	75% MIP	15% MIP	30 breaths/day	6 weeks
Kuo YC et al., 2017	EMST150, Aspire products	75% MEP	0% MEP	25 breaths/day (5 days/w)	5 weeks
Souza AKF et al., 2018	POWERbreathe classic light	50–60% MIP	20% MIP	90 breaths/day (7 days/w)	12 weeks
Erturk et al., 2020	IMT Threshold	30% MIP	No intervention	15 min twice a day (7 days/w)	12 weeks
Lin et al., 2020	IMT Threshold	30% MIP	NR	30–45 min/day (5 days/w)	12 weeks
Moawd et al., 2020	TRAINAIR^®^, Project Electronics Ltd., UK	75% MIP	≤10% MIP	120 breaths/day (3 days/w)	12 weeks
Nóbrega-Júnior et al., 2020	POWERbreathe classic light	50% MIP–2 weeks60% MIP–2 weeks75% MIP–4 weeks	0% MIP	180 breaths/day (7 days/w)	8 weeks
Ramos-Barrera et al., 2020	K3 series, POWERbreathe	75% MIP	15% MIP	30 breaths/day	6 weeks

Abbreviations: MIP: Maximum inspiratory pressure; MEP: Maximum expiratory pressure; NR: Not reported.

## Data Availability

Not applicable.
